# The effect of on-shelf sugar labeling on beverage sales in the supermarket: a comparative interrupted time series analysis of a natural experiment

**DOI:** 10.1186/s12966-021-01114-x

**Published:** 2021-04-06

**Authors:** J. C. Hoenink, J. M. Stuber, J. Lakerveld, W. Waterlander, J. W. J. Beulens, J. D. Mackenbach

**Affiliations:** 1 Department of Epidemiology and Data Science, Amsterdam Public Health research institute, Amsterdam UMC, Vrije Universiteit Amsterdam, De Boelelaan 1117, Amsterdam, the Netherlands; 2 Upstream Team, www.upstreamteam.nl, Amsterdam UMC, Vrije Universiteit Amsterdam, De Boelelaan 1117, Amsterdam, the Netherlands; 3 Department of Public and Occupational Health, Amsterdam Public Health research institute, Amsterdam UMC, University of Amsterdam, Meibergdreef 9, Amsterdam, the Netherlands; 4 Julius Center for Health Sciences and Primary Care, University Medical Center Utrecht, Utrecht University, Universiteitsweg 100, Utrecht, the Netherlands

**Keywords:** Nutritional labeling, Sugar-sweetened beverages, Grocery store, Interrupted time series analysis, Natural experiment

## Abstract

**Background:**

Nutrition labels show potential in increasing healthy food and beverage purchases, but their effectiveness seems to depend on the type of label, the targeted food category and the setting, and evidence on their impact in real-world settings is limited. The aim of this study was to evaluate the effectiveness of an industry-designed on-shelf sugar label on the sales of beverages with no, low, medium and high sugar content implemented within a real-world supermarket.

**Methods:**

In week 17 of 2019, on-shelf sugar labels were implemented by a Dutch supermarket chain. Non-alcoholic beverages were classified using a traffic-light labeling system and included the beverage categories “green” for sugar free (< 1.25 g/250 ml), “blue” for low sugar (1.25–6.24 g/250 ml), “yellow” for medium sugar (6.25–13.5 g/250 ml) and “amber” for high sugar (> 13.5 g/250 ml). Store-level data on beverage sales and revenue from 41 randomly selected supermarkets for 13 weeks pre-implementation and 21 weeks post-implementation were used for analysis. In total, 30 stores implemented the on-shelf sugar labels by week 17, and the 11 stores that had not were used as comparisons. Outcome measures were differences in the number of beverages sold in the four label categories and the total revenue from beverage sales in implementation stores relative to comparison stores. Analyses were conducted using a multiple-group Interrupted Time Series Approach. Results of individual store data were combined using random effect meta-analyses.

**Results:**

At the end of the intervention period, the changes in sales of beverages with green (B 3.4, 95%CI -0.3; 7.0), blue (B 0.0, 95%CI -0.6; 0.7), yellow (B 1.3, 95%CI -0.9; 3.5), and amber (B 0.9, 95%CI -5.5; 7.3) labels were not significantly different between intervention and comparison stores. The changes in total revenues for beverages at the end of the intervention period were also not significantly different between intervention and comparison stores.

**Conclusion:**

The implementation of an on-shelf sugar labeling system did not significantly decrease unhealthy beverage sales or significantly increase healthier beverage sales. Nutrition labeling initiatives combined with complementary strategies, such as pricing strategies or other healthy food nudging approaches, should be considered to promote healthier beverage purchases.

**Supplementary Information:**

The online version contains supplementary material available at 10.1186/s12966-021-01114-x.

## Introduction

Unhealthy dietary behavior such as a high intakes of sugars, saturated fats, and salt is associated with an increased risk of chronic diseases [1–3]. The World Health Organization has endorsed the need to create a supportive food environment by introducing interpretive and consumer friendly front-of-package (FOP) and/or on-shelf nutrition labels as a priority policy issue [4]. Recent systematic reviews concluded that FOP or on-shelf nutrition labels can be effective in increasing healthier product purchases or consumption [5–10].

The effectiveness of nutrition labels may vary according to the content of the label and the setting in which the label is applied. Content-wise, there are roughly two types of FOP or on-shelf nutrition labels: nutrient-specific and summary systems [11]. Nutrient-specific labels display one nutrient (e.g., single Traffic Light label) or a few key nutrients such as the percentage Guideline Daily Amounts (%GDA) or Multiple Traffic Lights (MTL). Summary systems use an algorithm to provide an overall nutritional score, which can be divided into summary icons that are either present or absent (e.g., the Keyhole symbol on healthy products) or summary icons displaying a graphic rating or numerical score such as the Health Star rating or Guiding Stars [11].

A systematic review comparing the effectiveness of various nutrition labels found that nutrient-specific labels are more effective in helping consumers to identify healthier products than summary systems [11]. Yet, a more recent review concluded that the results did not permit a verdict regarding whether nutrient-specific labels outperform summary labels [10]. The author did, however, conclude that the label designs that appear to be most successful are MTL labels, warning labels, and the Nutri-Score due to their easy to understand designs, and in the case of MTL and Nutri-Score the additional use of color [10]. Other studies also conclude that labels incorporating text with color to indicate levels of nutrients (e.g., MTL) are more effective than labels only displaying numeric information (e.g., %GDA) [11, 12]. These reviews did not distinguish between the placement (i.e., FOP or on-shelf labels) or between single nutrient and multiple nutrient labels (e.g., Traffic Light versus MTL) [10–12].

Another factor influencing the effectiveness of nutrition labels seems to be the setting in which they are assessed. Although studies have shown that nutrient-specific labels and summary systems are effective in increasing healthier purchases [5, 6], evidence suggests that real-world effect sizes are around 17 times smaller than those found in laboratory settings [13]. This difference may be explained by the fact that labels in real-world settings are generally less noticeable compared to laboratory settings, as they do not stand out much between the abundance of visual and auditory stimuli in real-world settings.

Nutrition labeling studies conducted in real-world settings include settings such as restaurants, sport-canteens, supermarkets, vending machines, and coffee shops [6, 7]. However, even these real-world settings may not be comparable due to the differences in terms of stocking, pricing and promotion of products. Nevertheless, a review investigating the real-world effectiveness of nutrient-specific labels and summary systems concluded that there was mixed evidence regarding the impact of nutrition labels on consumer purchases and highlighted a lack of studies that objectively measured food purchasing [12].

Supermarkets are an important real-world setting given that most foods are purchased here [14]. Two randomized control trials (RCTs) found that nutrient-specific and summary system labels delivered via a smartphone application were somewhat effective [15, 16], mainly in participants who used the labeling intervention more often than average users [16]. While RCTs deliver important evidence based on their high internal validity, RCTs may not accurately mimic real world conditions [17]. Therefore, evidence from natural experiments are also needed as these results are more generalizable. Six studies using data from experiments in supermarket settings showed mixed evidence regarding the effect of nutrient-specific FOP labels [18–20] and the on-shelf Guiding Star summary scheme [21–23] on food sales or purchases. These studies used various types of labels implemented across various food categories – potentially explaining the mixed results. Furthermore, only one study included a control condition [23]. High quality evidence on the effects of nutrition labelling on food purchases is thus scarce [10].

Many countries have mandatory nutrition facts panels on the backside of prepackaged food products. Some countries additionally have mandatory FOP nutrition labels on prepackaged food products (e.g., Chile and Finland) and other countries have voluntary industry-designed nutrition labels such as the Facts Up Front label in the United States and the supermarket color-coded on-shelf sugar labels across supermarket chains in the Netherlands [5, 18]. This study evaluated the effectiveness of such an industry-designed color-coded on-shelf sugar label (a type of nutrient-specific label) on the number of non-alcoholic beverage sales and revenue using a natural experimental design including comparison stores. As the effectiveness of nutrition labels seems to depend on the type of label [11, 12], the targeted food category [23] and the setting [13], this study contributes to a growing literature base investigating the impact of single nutrient-specific on-shelf nutrition label interventions in real-world supermarkets.

## Methods

This natural experimental study used sales data from a Dutch supermarket chain that implemented on-shelf sugar labels in their stores in 2019. Using a random number generator, we retrospectively selected fifty stores. We categorized stores that successfully implemented the on-shelf sugar labels as intervention stores and used the remaining stores as comparisons in an interrupted time series analysis. We hypothesized that the on-shelf sugar labels increased the sales of sugar free and low sugar beverages, while decreasing the sales of high sugar beverages.

### The on-shelf sugar labels

**Fig. 1:**
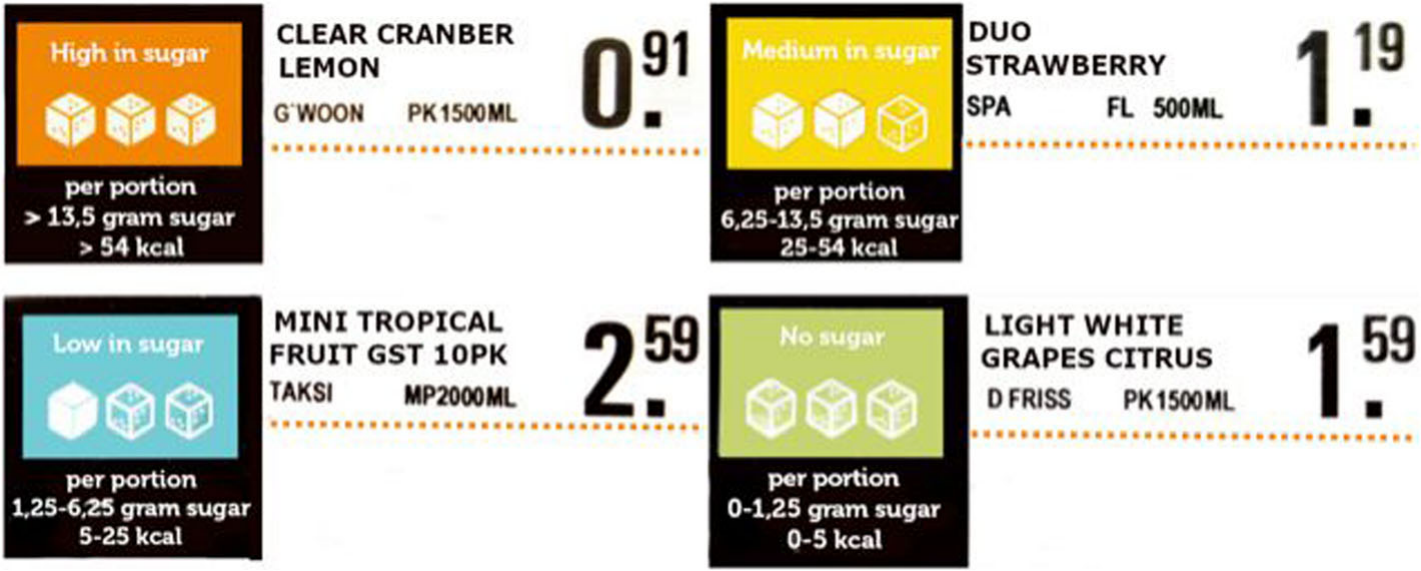
Examples of the on-shelf sugar labels placed next to the product descriptions and price tags in Euros (translated from Dutch to English)

All non-alcoholic beverages such as sodas, energy drinks, juices and water were labeled using a nutrient-specific traffic-light labeling system. Milk-based beverages were excluded. The graphical lay-out of the labeling system was designed following the corporate identity of the supermarket chain. The content of traffic-light labeling system was designed by the supermarket chain based on the Nutri-Score label [24], the Evolved Nutrition Label (developed by several companies from the food industry), and input from their costumer panel. The categories consisted of “green” for sugar free beverages (< 1.25 g/250 ml), “blue” for low sugar beverages (1.25–6.24 g/250 ml), “yellow” for medium sugar beverages (6.25–13.5 g/250 ml) and “amber” for high sugar beverages (> 13.5 g/250 ml). Categorization of green and blue beverages was based on the legal rules for nutrition claims as defined by the European Commission [25]. The categorization of yellow and amber beverages was based on the Evolved Nutrition Label, combined with the Nutri-Score guidelines [24]. The on-shelf sugar labels were displayed next to an individual price tag (Fig. 1). Such placement of on-shelf labels next to the price tag is commonly used by the supermarket chains to highlight additional information on a specific product (e.g., to highlight store brand products). The on-shelf sugar labels displayed, besides the traffic-light colors, also the range in numerical sugar content (in grams per 250 ml portions) with an additional image of the number of sugar cubes. In addition, the shelf included a small poster explaining the meaning of the on-shelf sugar labels (Supplementary Figure 1; including English translation). Supplementary Figure 2 displays a photograph of the on-shelf sugar labels implemented in store.

### Store selection

The supermarket chain headquarters notified all their stores to implement the on-shelf sugar labels in week 17 of 2019. The supermarket chain has three types of stores in the Netherlands: regular supermarkets, compact stores and city stores. Compact stores are small regular supermarkets, whereas city stores are small supermarkets with a different pricing line and a larger focus on convenience products. The researchers randomly selected 50 out of the possible ~ 300 stores to be included in the study. Weekly sales data were obtained from January 2019 to August 2019 for the four beverages categories (i.e., green, blue, yellow, and amber), resulting in data from 14 weeks before implementation and 21 weeks after the implementation of the sugar shelf labels. Literature has indicated to include a minimum number of 3 to 10 time-points in order to have enough power [26].

Due to temporary closure for store renovations, five of the selected stores did not include sales data for the entire study period and were therefore excluded from analysis. In weeks 20 to 22, the supermarket chain evaluated the implementation fidelity of the on-shelf sugar labels by asking the store managers to report back on whether the labels had been implemented. Of the 45 remaining stores, 29 stores had implemented the on-shelf sugar labels by weeks 20 to 22. We contacted the remaining 16 stores in January 2020 to check whether the on-shelf sugar labels had been implemented by then. One store indicated to have implemented the on-shelf sugar labels before the summer of 2019 and was included as intervention store. Out of the remaining stores, 10 had not implemented the on-shelf sugar labels as of January 2020, and one store had implemented the labels in the fall of 2019. These 11 stores were included as comparison stores. The last four stores indicated to have implemented the on-shelf sugar labels, but were unsure of the exact time period and were thus excluded from the analyses.

Ultimately, we included the 30 stores that implemented the on-shelf sugar labels shortly after week 17 and had data for all time-points as intervention stores, and included the 11 stores that did not implement the on-shelf sugar labels during the study period and had data for all time-points as comparison stores.

### Outcome measures

Weekly sales data were extracted for the four beverage categories. The primary outcomes were changes in the number of non-alcoholic green, blue, yellow, and amber beverages sold (excluding products on sale), and changes in the total revenue of beverage sales. Secondary outcomes were changes in the revenue of beverages for each of the four beverage groups (excluding products on sale) and total sugar content of beverages sold. These outcomes were chosen as they capture the direct impact of targeted beverages on beverage purchases, the indirect effect on other beverage purchases, the impact on excess sugar intake from beverage purchases and the impact on beverage revenue [27]. Additionally, outcomes based on both volume (i.e., sales-by-quantity) and revenue give more confidence regarding the effect estimate of on-shelf sugar labels.

Whereas outcomes involving the volume of beverages sold is of interest from a public health perspective, it can mask or pronounce effects if for example the average pack size bought changes over time. On the other hand, using revenue as an outcome avoids this challenge but may introduce problems if the price or relative price of beverages compared to other items changes over the study period. By examining patterns for both outcomes and checking for consistency, we can be more confident that there is a genuine underlying effect [28].

### Statistical analysis

For the main analyses we used a comparative interrupted time-series (CITS) analysis as it is considered the most suitable approach to evaluate natural experimental data where researchers have no control over the design and delivery of the intervention [29–31]. Given that 11 stores had not implemented the sugar shelf-labels during the study period, we were able to include those as comparison stores [32]. Compared to single-group interrupted time series analyses (ITSA), the addition of a comparison group allows for a more reliable estimation of the impact of the intervention [33].

In the single-group ITSA models, the trend in beverage sales within the pre-implementation period is carried on in the post-implementation period as the counterfactual of what is expected to happen if the on-shelf sugar labels were never implemented [34]. Estimates of both the change in ‘level’ and ‘trend’ of the observed versus counterfactual regression lines are calculated. The level change is the difference in intercepts between the regression lines estimated from observations before and after the interruption, whereas the trend change is the difference in slopes. In CITS models, the counterfactual not only includes information on what would have been expected to happen had the intervention not occurred from both the pre-implementation data in the intervention case, but it also includes the difference in post-implementation slopes between the intervention and comparison stores [34]. The counterfactual is compared to a similar regression line calculated from the observation of what did happen following the implementation of the on-shelf sugar labels.

Using the package ‘ITSA’ in STATA 14.0 [35], changes in non-alcoholic beverage sales associated with the implementation of the sugar shelf-labels as an immediate change in the number of beverages sold were modelled. The step change was defined as a categorical variable equal to zero before implementation (week 18) and one after implementation of the on-shelf sugar labels, leading to 14 weekly pre-implementation time-points and 21 weekly post-implementation time-points. This approach assumes an immediate and stable effect of the intervention.

We used the STATA package ITSAMATCH to match comparison stores based on the pre-intervention level and trend of beverage sales in the intervention stores [36]. Comparison stores were matched for each intervention store for each individual outcome measure. A suitable comparison store is one with a similar pre-implementation curve, but not necessarily at a similar level [34]. If ITSAMATCH did not find a suitable comparison store, we selected comparison stores with similar characteristics and assessed whether these were suitable comparisons based on the similarity of pre-implementation curves (*p*-value curve > 0.20) [36]. Suitable comparison stores were matched to intervention stores within all analyses based on pre-intervention level and trend of beverage sales, with the exception of two stores for the volume sales changes in green beverages where no suitable comparison store with similar sales patterns was found. Therefore, those two stores were excluded from the analyses, resulting in 28 instead of 30 stores analyzed for this outcome.

Separate analyses for each intervention store were conducted as we expected the supermarkets to have different customer bases, to have implemented the on-shelf sugar labels at different time points, and because different comparison stores were appropriate for different intervention stores [34]. Then, a meta-analytical approach was used to obtain an overall estimate of the effect, to explore the consistency of the effect size across individual supermarkets, and to identify whether outcomes varied when stratified by store characteristics [27]. In case of substantial heterogeneity (I^2^ > 50%), subgroup analyses were conducted by store type (i.e., regular, compact, or city store), the region where the store is located and store area level deprivation.

All models were adjusted for temperature-driven variability in the consumption of beverages by including a variable representing the average of the highest measured weekly daytime temperature over the study period to adjust for changes in beverage sales by season. Although adjustment was not necessary for the CITS models, we did so to facilitate direct comparison with the ITSA model. The models were fitted assuming an autoregressive correlation with varying lags (depending on the stores and outcomes), using the Newey-West estimation method. A random-effects meta-analysis approach was then used to obtain a pooled effect estimate.

### Sensitivity analysis

We carried out sensitivity analyses to examine the model assumptions. Firstly, all analyses conducted for the primary outcomes were repeated, but using a single-group ITSA design, i.e., excluding the comparison stores. Conducting both multiple- and single-group ITSA provides additional insights into possible history bias or changes in the comparison group but not the treatment group [33]. Secondly, the robustness of the timing of the effect was investigated by assuming the date of implementation to be in week 22 instead of 18 to allow for delayed implementation effects. Finally, all analyses conducted for the primary outcome were repeated including non-alcoholic beverages that were on sale during the study period.

## Results

Eighty percent of the included stores were regular supermarkets and approximately 50% of stores were located in socially deprived areas (Supplementary Table 1). Compact and city stores were relatively more often included as comparison stores compared to the intervention stores (35% versus 15%). After the implementation of the on-shelf sugar labels, the mean weekly sales of all four beverage categories increased in almost all intervention and comparison stores (Table 1). Also, the mean weekly revenue on all beverage sales increased in all stores. Amber beverages had the highest number of sales.

**Table 1:** Mean weekly number of beverages sold and total beverage revenue before and after the implementation of on-shelf sugar labels for all supermarkets

	Weekly mean (SD) sales of amber sugar beverages		Weekly mean (SD) sales of yellow sugar beverages		Weekly mean (SD) sales of blue sugar beverages		Weekly mean (SD) sales of green free beverages		Weekly mean (SD) revenue from all beverages in €	
Supermarket number	Pre-implementation	Post-implementation	Pre-implementation	Post-implementation	Pre-implementation	Post-implementation	Pre-implementation	Post-implementation	Pre-implementation	Post-implementation
	Intervention stores									
1	1203 (189)	1440 (220)	269 (51)	357 (63)	93 (17)	107 (20)	785 (119)	1032 (245)	2779 (420)	3453 (597)
2	1774 (205)	1942 (220)	495 (79)	650 (105)	141 (24)	158 (26)	983 (129)	1222 (235)	4720 (577)	5562 (719)
3	2765 (297)	2940 (330)	654 (103)	776 (152)	235 (34)	231 (38)	1160 (145)	1396 (255)	5630 (704)	6268 (887)
4	1599 (212)	1745 (311)	339 (70)	365 (65)	119 (36)	149 (45)	462 (82)	607 (151)	2334 (311)	2754 (535)
5	867 (184)	1052 (161)	242 (42)	309 (63)	109 (24)	126 (26)	430 (47)	569 (133)	2432 (355)	3000 (505)
6	940 (111)	1036 (115)	247 (39)	306 (54)	81 (8)	89 (18)	654 (103)	829 (164)	2526 (330)	2977 (443)
7	2203 (294)	2693 (731)	545 (114)	809 (290)	90 (18)	114 (22)	1187 (232)	1759 (627)	5000 (653)	6264 (1572)
8	2398 (267)	2694 (333)	673 (112)	815 (134)	485 (106)	550 (161)	946 (117)	1232 (196)	5729 (599)	6786 (989)
9	1285 (137)	1531 (152)	374 (39)	466 (72)	117 (12)	142 (24)	897 (105)	1057 (154)	3949 (444)	4572 (514)
10	1773 (225)	1879 (219)	547 (70)	689 (137)	248 (49)	264 (51)	760 (133)	935 (151)	3892 (504)	4399 (569)
11	2434 (278)	2588 (389)	664 (78)	802 (181)	427 (62)	452 (70)	1315 (175)	1595 (295)	6839 (786)	7762 (1176)
12	901 (75)	1061 (120)	301 (44)	400 (63)	101 (20)	103 (22)	648 (64)	864 (153)	2290 (185)	2892 (384)
13	2823 (202)	2721 (497)	566 (81)	692 (173)	254 (28)	269 (55)	954 (70)	1099 (257)	4723 (315)	5126 (922)
14	1050 (157)	1185 (183)	342 (46)	459 (86)	134 (15)	168 (40)	612 (94)	825 (190)	3020 (463)	3756 (653)
15	3179 (305)	3049 (250)	667 (111)	706 (105)	187 (31)	193 (34)	1293 (156)	1434 (206)	5370 (595)	5457 (562)
16	682 (113)	884 (165)	190 (45)	267 (62)	114 (25)	143 (28)	308 (50)	470 (111)	1759 (335)	2451 (442)
17	688 (104)	768 (98)	240 (51)	310 (42)	78 (16)	80 (18)	432 (75)	568 (110)	1836 (303)	2280 (329)
18	2956 (204)	3181 (337)	686 (82)	807 (150)	232 (22)	284 (37)	1089 (142)	1370 (243)	5387 (414)	6274 (803)
19	598 (65)	670 (115)	155 (44)	225 (71)	19 (5)	30 (10)	426 (51)	618 (155)	1695 (195)	2070 (393)
20	2870 (227)	3358 (444)	696 (92)	957 (189)	293 (40)	329 (50)	1215 (183)	1578 (294)	5290 (534)	6563 (980)
21	2202 (215)	2652 (365)	425 (115)	669 (176)	55 (14)	74 (23)	827 (195)	1357 (442)	4418 (658)	5955 (1256)
22	738 (70)	857 (146)	244 (37)	334 (87)	92 (17)	92 (20)	461 (67)	581 (117)	2092 (194)	2472 (469)
23	1497 (191)	1645 (232)	364 (69)	459 (93)	237 (40)	231 (49)	734 (71)	885 (188)	3975 (385)	4410 (627)
24	1423 (300)	1975 (429)	446 (91)	802 (262)	224 (59)	336 (96)	806 (214)	1396 (422)	4117 (931)	6224 (1493)
25	870 (128)	1053 (145)	248 (85)	321 (88)	118 (17)	151 (29)	536 (121)	755 (185)	2523 (428)	3287 (581)
26	510 (79)	583 (109)	154 (35)	207 (59)	36 (7)	54 (14)	325 (73)	501 (144)	1596 (272)	2031 (437)
27	880 (126)	1012 (159)	370 (47)	443 (95)	111 (16)	130 (29)	479 (52)	638 (159)	2487 (319)	2985 (513)
28	1848 (210)	1906 (304)	352 (41)	475 (90)	122 (15)	135 (30)	887 (86)	1069 (223)	3800 (343)	4304 (666)
29	1063 (160)	1455 (307)	259 (64)	435 (140)	239 (67)	322 (100)	519 (108)	913 (340)	3046 (468)	4521 (1143)
30	620 (79)	785 (122)	166 (46)	244 (74)	48 (12)	57 (15)	418 (132)	539 (155)	1806 (307)	2417 (472)
	Control stores									
31	1200 (131)	1411 (190)	351 (44)	491 (100)	163 (23)	166 (39)	604 (71)	770 (184)	2862 (242)	3364 (499)
32	1284 (156)	1734 (361)	232 (42)	323 (98)	23 (7)	99 (43)	527 (114)	862 (254)	2844 (271)	3919 (884)
33	1191 (128)	1216 (255)	271 (51)	346 (104)	81 (19)	100 (33)	501 (63)	685 (168)	2219 (272)	2589 (597)
34	1066 (94)	1184 (165)	291 (77)	349 (74)	122 (22)	134 (34)	455 (93)	629 (166)	2455 (283)	2857 (511)
35	2640 (192)	2630 (278)	444 (90)	496 (59)	129 (18)	139 (22)	782 (91)	1012 (203)	4510 (406)	4805 (560)
36	1473 (163)	1739 (294)	390 (83)	572 (153)	177 (37)	225 (60)	655 (103)	911 (255)	3577 (458)	4608 (945)
37	446 (43)	429 (71)	105 (21)	116 (27)	35 (8)	34 (10)	207 (25)	250 (75)	989 (61)	1054 (196)
38	2161 (222)	2277 (292)	639 (84)	742 (138)	214 (37)	220 (44)	1583 (216)	1851 (306)	5899 (700)	6497 (912)
39	1227 (183)	1438 (176)	368 (50)	449 (97)	212 (56)	275 (77)	573 (99)	772 (179)	3266 (466)	4147 (606)
40	1747 (245)	1927 (315)	425 (86)	514 (93)	213 (56)	242 (62)	684 (85)	998 (349)	4098 (581)	5057 (760)
41	357 (52)	421 (124)	96 (18)	112 (39)	48 (10)	54 (12)	95 (14)	143 (57)	778 (92)	1011 (326)

Abbreviations: *SD* Standard Deviation

### Main findings

**Fig. 2:**
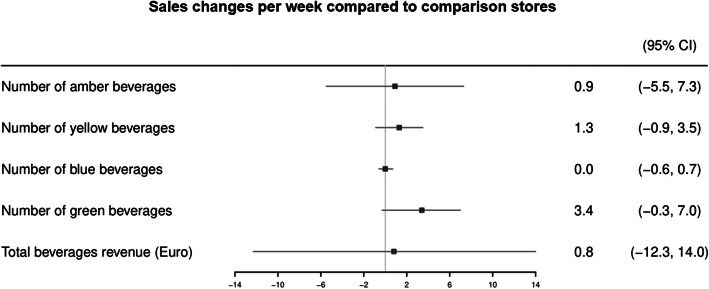
Pooled changes in beverage sales and total beverage revenue compared to comparison stores

After the implementation of on-shelf sugar labels, amber beverage sales slightly increased by 0.9 (95%CI -5.5; 7.3), yellow beverage sales slightly increased by 1.3 (95%CI -0.9; 3.5) and green beverage sales slightly increased by 3.4 (95%CI -0.3; 7.0) units per week following the implementation compared to the counterfactual (i.e., the comparison stores) (Fig. 2). These point estimates suggest a slight increase in amber, yellow and green beverage sales, however, the lower limit of the 95% confidence intervals suggest that the effects may also be negative. Implementation of on-shelf sugar labels did not change the sales of blue beverages (B 0.0, 95%CI -0.6; 0.7). Total beverage revenue only increased by 0.8 (95%CI -12.3; 14.0) Euros per week following the implementation compared to the counterfactual.

Changes in beverage sales and total revenue on store-level can be found in Supplementary Figures 3 and 4. Only for one individual intervention store, a statistically significant increase of 18.8 (95%CI 5.4; 32.1) units of green beverage sales per week following the implementation compared to the counterfactual was found. No other statistically significant changes on store-level were found for the primary outcome measures. Given the low heterogeneity between stores, subgroup analyses were not conducted.

Regarding the secondary outcomes, the amount of sugar purchased slightly increased by 2.4 (95%CI -3.5; 8.3) units per week from beverage sales following the implementation compared to the counterfactual (Supplementary Figure 5). Meta-analyzed results showed that the revenue on green beverages increased statistically significantly by 4.1 (95%CI 0.1; 8.0) Euros (or US$ 4.9) per week following the implementation compared to the counterfactual (Supplementary Figure 6d). No noteworthy changes in the revenue of the other three beverage categories were found (Supplementary Figures 6a, 6b and 6c).

### Sensitivity analyses

Analyses where the pre-implementation trend was used as the counterfactual without including comparison stores indicated that the green, blue and yellow beverage sales increased by 9.2 (95%CI 6.6; 11.7), 0.7 (95%CI 0.2; 1.1) and 4.8 (95%CI 2.8; 6.9) units per week following the implementation compared to the pre-implementation trend, respectively (Supplementary Figure 7). The total revenue from all beverages sold increased by 25.3 (95%CI 16.0; 34.6) Euros (or US$ 30.1) per week following the implementation compared to the pre-implementation trend (Supplementary Figure 7). Only the sales of amber beverages did not change significantly.

Regarding the robustness of the timing of the effect, changing the timing of the on-shelf sugar labels to week 22 instead of week 18 did not affect the results (Supplementary Figure 8). Results were similar when including the sales data of beverages on sale (Supplementary Figure 9).

## Discussion

This study used supermarket sales data to investigate the effect of an industry-designed on-shelf nutrient-specific labeling system on beverage sales and revenue. Our results indicate that the implementation of on-shelf sugar labels does not significantly change the beverage sales between intervention and comparison stores in all four beverage categories (i.e., green, blue, yellow, and amber) nor on total beverage sales revenue.

Real-world effects of nutrition labeling in the supermarket were examined previously [15, 16, 18–23], but a comparison of study findings is not straight forward. The methodological designs (natural experiment or randomized controlled trial), analytic approaches (CITS, ITSA, or between group comparison), the placement of the nutrition labels (FOP, on-shelf or via a mobile app), the type of labels (nutrient-specific or summary system), and the food categories on which the labels are implemented all vary across studies.

Hobin et al. (2017), a study most comparable to the current study, investigated the effect of nutrition labeling on beverage sales using CITS analysis. This study found, contrary to our results, a statistically significant decrease of 2.6% in unhealthy beverage sales compared to the control stores [23]. Similar to our results, the healthier beverages sales did not significantly change. The somewhat different results between the aforementioned study and the current study may be explained by the fact that the study by Hobin et al. (2017) used an on-shelf summary system (Guiding Stars) [23], while the current study evaluated an on-shelf nutrient-specific label. Other real-world studies investigating the effect of nutrition labeling in the supermarket did not include a control condition [20–22], and/or investigated other food categories [19–21], or used nutritional warning labels [18].

In line with our findings, Sacks et al. (2009) showed no beneficial effects on the healthiness of sold products after the implementation of a FOP nutrient-specific system on ready-to-eat meals and sandwiches [20]. Cawley et al. (2015) examined the effect of an on-shelf summary system (Guiding Stars) in the supermarket. Although the unhealthy beverages decreased by 27%, similar results were observed for the beverage sales with any number of stars. Likewise, Sutherland et al. (2010) showed that the Guiding Stars system significantly decreased the sales of zero-star products [21]. However, given that neither studies included comparison stores, the sale changes may have been driven by overall changes over the study period. Especially in the case of Cawley et al. (2015), where a decrease of beverage sales was found in all beverage categories [22].

As demonstrated by our study, the absence of comparison data can largely affect the outcomes. When we excluded the comparison stores and only compared the post-implementation trend with the pre-implementation trend for intervention stores as the counterfactual, our sensitivity analyses showed statistically significant, albeit small, increases of green, blue and yellow beverage sales after the implementation of on-shelf sugar labels. Comparing our ITSA results with the CITS results shows that including comparison stores is necessary in order to account for possible trends in beverage sales not attributable to the implementation of the on-shelf sugar labels. However, given that this study used a natural experimental design and not an RCT, the comparison stores used in this study were not randomly selected. Also, issues with implementation fidelity might attenuate the observed results. RCTs would be needed to allow for random intervention allocation, and for monitoring implementation fidelity, while the data from this natural experiment may more accurately reflect effect sizes after the implementation of nutrition labeling under normal circumstances (e.g., when not under supervision by researchers). Two previous studies investigated implementation of nutrition labels via a smartphone application after scanning product barcodes [15, 16]. One of these studies observed that among consumers who frequently used the application to receive the nutrition labeling (summary system) healthier beverage sales significantly increased [16]. The other study found that the nutritional value of purchased foods was healthier after the implementation of one out of the five of the tested nutrition labels (i.e., a nutrition information panel including a recommendation or warning) [15].

Based on the current evidence it seems that nutrition labeling alone would not be sufficient to increase healthier product sales and/or decrease unhealthy product sales. Implementing multiple strategies targeted at discouraging unhealthy and/or encouraging healthier product sales are likely needed. Besides governmental guidelines on a mandatory, consistent, and easily interpretable labeling systems – which can inform consumers on healthier choices and stimulate product reformulation in the industry [37] – a supplementary strategy could include taxing of amber and yellow beverages to discourage purchases and should also stimulate product reformulation [38–40]. The tax revenue could in turn be used to encourage healthy products by subsidizing these. Furthermore, nutrition labeling initiatives can be considered information nudges [41]. Other nudging strategies can additionally be used to promote green and blue beverages purchases. Nudges are various environmental changes that promote healthier choices without removing the unhealthier choices [42]. Examples of nudges are placing healthy beverages at eye-level and enhancing their visibility using attractive promotion materials, vivid product descriptions, or increasing healthy beverage availability [41, 43]. A recent study indeed showed that combining multiple strategies within the supermarket setting is important, as nudges alone did not increase healthy food purchases, while combining pricing strategies (i.e., taxing unhealthy products and subsidizing healthy products) with nudges had the largest impact on healthier purchasing behaviors [44]. Nutrition labeling initiatives combined with complementary strategies should therefore be implemented and evaluated across multiple food groups within the supermarket to promote a shift towards a healthy dietary pattern [45]. Moreover, research is needed on the potential long-term effectiveness of these strategies and their potential to improve population health.

This study has several strengths. We analyzed the effect of on-shelf nutrient-specific labeling on beverage purchases using real-world supermarket sales data including comparison stores. Therefore, this study generated evidence relevant for real world implementation. Moreover, we used relevant business outcomes in order to investigate the sustainability of this retail-led health intervention. We analyzed our data with a CITS approach which is the recommended approach to analyze these types of natural experiments [46, 47]. With the inclusion of comparison stores we accounted for possible time-varying confounders [33]. Also, we had access to 34 time-points whereas literature indicates as a rule of thumb that the minimum number of time points required for this type of analysis lies between 3 to 10 [26]. Furthermore, pooling of overall effects using a meta-analysis approach increased statistical power of the analysis and improved overall interpretation of the findings. Lastly, the results can be generalizable to other supermarkets in the Netherlands using color-coded on-shelf sugar labels on non-alcoholic beverages given the inclusion of a large and diverse selection of supermarket stores.

Despite these strengths, some limitations need to be considered. Natural experiments do not allow for regulation of the intervention development, allocation and implementation by researchers. Indeed, comparison stores were self-selected and probably had reasons for not implementing the on-shelf sugar labels which remain unknown to the researchers. This could for example explain why there were more compact stores in the comparison group compared to the intervention group*.* Moreover, due to the nature of this study, we could not study consumer awareness of the labels and did not have access to detailed information on implementation fidelity and could therefore not account for that in the analyses. Similar to other studies [20, 22, 23], we modelled changes in beverage sales associated with the implementation of the sugar shelf-labels as an immediate step change in the number of beverages sold. This approach assumes an immediate and stable effect of the on-shelf labels directly after implementation, whereas in practice the effectiveness of labels may increase gradually over time. However, since our sensitivity analysis of changing the timing of the implementation of the on-shelf sugar labels did not show different the results, we have no indications for a gradual increase of effectiveness over time.

## Conclusion

This study provides important evidence from a natural experiment in a real-world supermarket setting regarding the effectiveness of an industry-designed on-shelf sugar label on beverage sales and revenue. The implementation of an on-shelf sugar labeling system did not significantly decrease unhealthy beverage sales and also did not significantly increase sales of beverages labeled as healthier. Nutrition labeling initiatives combined with complementary strategies, such as pricing strategies or other healthy food nudging approaches, should be considered to promote healthier beverage purchases.

## Supplementary Information

**Additional file 1.** Online supplementary materials.

## Data Availability

The data analyzed during the current study are not publicly available as a result of a confidentiality agreement with our supermarket partner. The analysis plan and syntax are available from the corresponding author on reasonable request.

## Abbreviations

CITS: Comparative interrupted time-series
FOP: Front-of-package
ITSA: Interrupted time-series analyses
