# Supplementary material for: The effect of on-shelf sugar labeling on beverage sales in the supermarket: a comparative interrupted time series analysis of a natural experiment
Source: Int J Behav Nutr Phys Act. 2021 Apr 6;18:49. doi: 10.1186/s12966-021-01114-x (PMC8025575; doi:10.1186/s12966-021-01114-x)
Supplement: Supplementary file 1 — Additional file 1. Online supplementary materials. [file 12966_2021_1114_MOESM1_ESM.docx]

**The effect of on-shelf sugar labelling on beverage sales in the supermarket:**

**an interrupted time series analysis of a natural experiment**

*Online supplementary material*

Jody C. Hoenink & Josine M. Stuber et al.

[Supplementary Table 1. Supermarket characteristics 2](#_Toc65150510)

[Supplementary Figure 1. In-store poster explaining the on-shelf sugar labelling colours in Dutch, including English translation 3](#_Toc65150511)

[Supplementary Figure 2. Photograph of the on-shelf sugar labels implemented in store 4](#_Toc65150512)

[Supplementary Figure 3a. Change in sales of amber beverages compared to comparison stores 5](#_Toc65150513)

[Supplementary Figure 3b. Change in sales of yellow beverages compared to comparison stores 6](#_Toc65150514)

[Supplementary Figure 3c. Change in sales of blue beverages compared to comparison stores 7](#_Toc65150515)

[Supplementary Figure 3d. Change in sales of green beverages compared to comparison stores 8](#_Toc65150516)

[Supplementary Figure 4. Change in beverage revenue compared to comparison stores 9](#_Toc65150517)

[Supplementary Figure 5. Change in total sugar from beverages sold compared to comparison stores 10](#_Toc65150518)

[Supplementary Figure 6a. Change in revenue after implementation of on-shelf sugar labels of amber beverages compared to comparison stores 11](#_Toc65150519)

[Supplementary Figure 6b. Change in revenue after implementation of on-shelf sugar labels of yellow beverages compared to comparison stores 12](#_Toc65150520)

[Supplementary Figure 6c. Change in revenue after implementation of on-shelf sugar labels of blue beverages compared to comparison stores 13](#_Toc65150521)

[Supplementary Figure 6d. Change in revenue after implementation of on-shelf sugar labels of green beverages compared to comparison stores 14](#_Toc65150522)

[Supplementary Figure 7. Overall pooled change in sales after implementation of on-shelf sugar labels of amber, yellow, blue and green beverages and change in total beverage revenue 15](#_Toc65150523)

[Supplementary Figure 8. Overall pooled change in sales after implementation of on-shelf sugar labels of amber, yellow, blue and green beverages and total revenue compared to comparison stores, when implementation timing is set at week 22 instead of week 18 16](#_Toc65150524)

[Supplementary Figure 9. Overall pooled change in sales after implementation of on-shelf sugar labels of amber, yellow, blue and green beverages and total revenue compared to comparison stores, including beverages on sale 17](#_Toc65150525)

# Supplementary Table 1. Supermarket characteristics

| Supermarket number | Store size (m^2^) | Type of store^a^ | Area deprivation^b^ |
| --- | --- | --- | --- |
| Intervention stores | | | |
| Supermarket 1 | 870 | Regular | Low |
| Supermarket 2 | 1164 | Regular | Low |
| Supermarket 3 | 1359 | Regular | High |
| Supermarket 4 | 505 | Regular | High |
| Supermarket 5 | 956 | Regular | Low |
| Supermarket 6 | 838 | Regular | Low |
| Supermarket 7 | 443 | Regular | Low |
| Supermarket 8 | 1128 | Regular | High |
| Supermarket 9 | 1263 | Regular | High |
| Supermarket 10 | 1144 | Regular | High |
| Supermarket 11 | 1521 | Regular | High |
| Supermarket 12 | 1235 | Regular | Low |
| Supermarket 13 | 896 | Regular | High |
| Supermarket 14 | 1109 | Regular | High |
| Supermarket 15 | 740 | Regular | High |
| Supermarket 16 | 757 | Regular | Low |
| Supermarket 17 | 801 | Regular | Low |
| Supermarket 18 | 811 | Regular | High |
| Supermarket 19 | Missing | City store | Low |
| Supermarket 20 | Missing | Regular | High |
| Supermarket 21 | 524 | City store | Low |
| Supermarket 22 | Missing | Regular | Low |
| Supermarket 23 | Missing | Regular | High |
| Supermarket 24 | 1023 | Regular | High |
| Supermarket 25 | 760 | Regular | Low |
| Supermarket 26 | 624 | Compact | Low |
| Supermarket 27 | 792 | Regular | Low |
| Supermarket 28 | 838 | Regular | Low |
| Supermarket 29 | 754 | Regular | Low |
| Supermarket 30 | 619 | Compact | Low |
| Comparison stores | | | |
| Supermarket 31 | 1036 | Regular | High |
| Supermarket 32 | Missing | City store | High |
| Supermarket 33 | Missing | Regular | High |
| Supermarket 34 | Missing | Regular | Low |
| Supermarket 35 | 169 | Compact | High |
| Supermarket 36 | 1093 | Regular | High |
| Supermarket 37 | 273 | Compact | Low |
| Supermarket 38 | 1131 | Regular | Low |
| Supermarket 39 | 1010 | Regular | Low |
| Supermarket 40 | 916 | Regular | Low |
| Supermarket 41 | 427 | Compact | High |

^a^Regular: Regular supermarket, Compact: small regular supermarket, City Store: small city supermarket with different pricing line;

^b^High or low area deprivation level as compared to the national average.

|  |  |
| --- | --- |
| 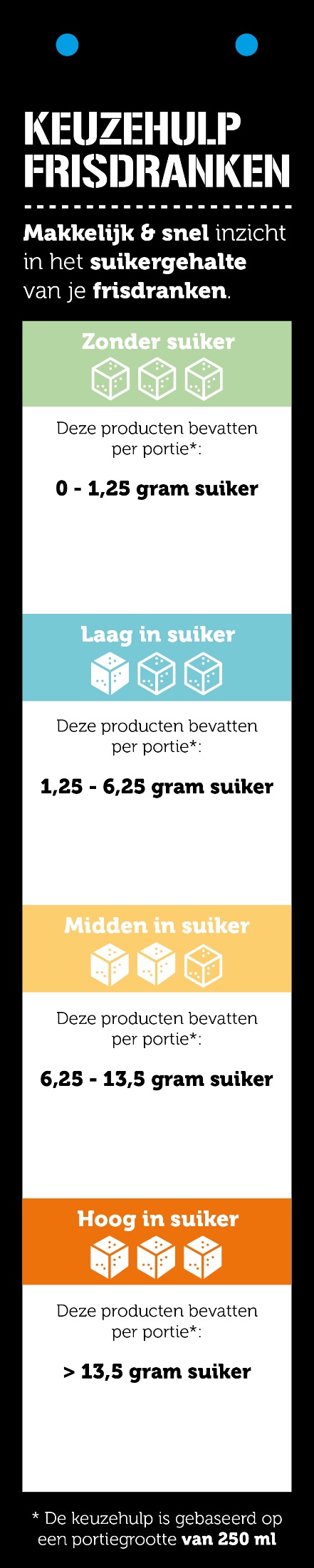 | ’Beverages decision aid’  ‘Easy and quick insight into the sugar content of your beverages.’  ‘No sugar’  ‘These products contain per serving*:’  ‘0-1.25 gram sugar’  ‘Low in sugar’  ‘These products contain per serving*:’  ‘1.25-6.25 gram sugar’  ‘Medium in sugar’  ‘These products contain per serving*:’  ‘6.25-13.5 gram sugar’  ‘High in sugar’  ‘These products contain per serving*:’  ‘>13.5 gram sugar’  ‘* This discicion aid is based on serving sizes of 250 ml’ |

# Supplementary Figure 1. In-store poster explaining the on-shelf sugar labelling colours in Dutch, including English translation


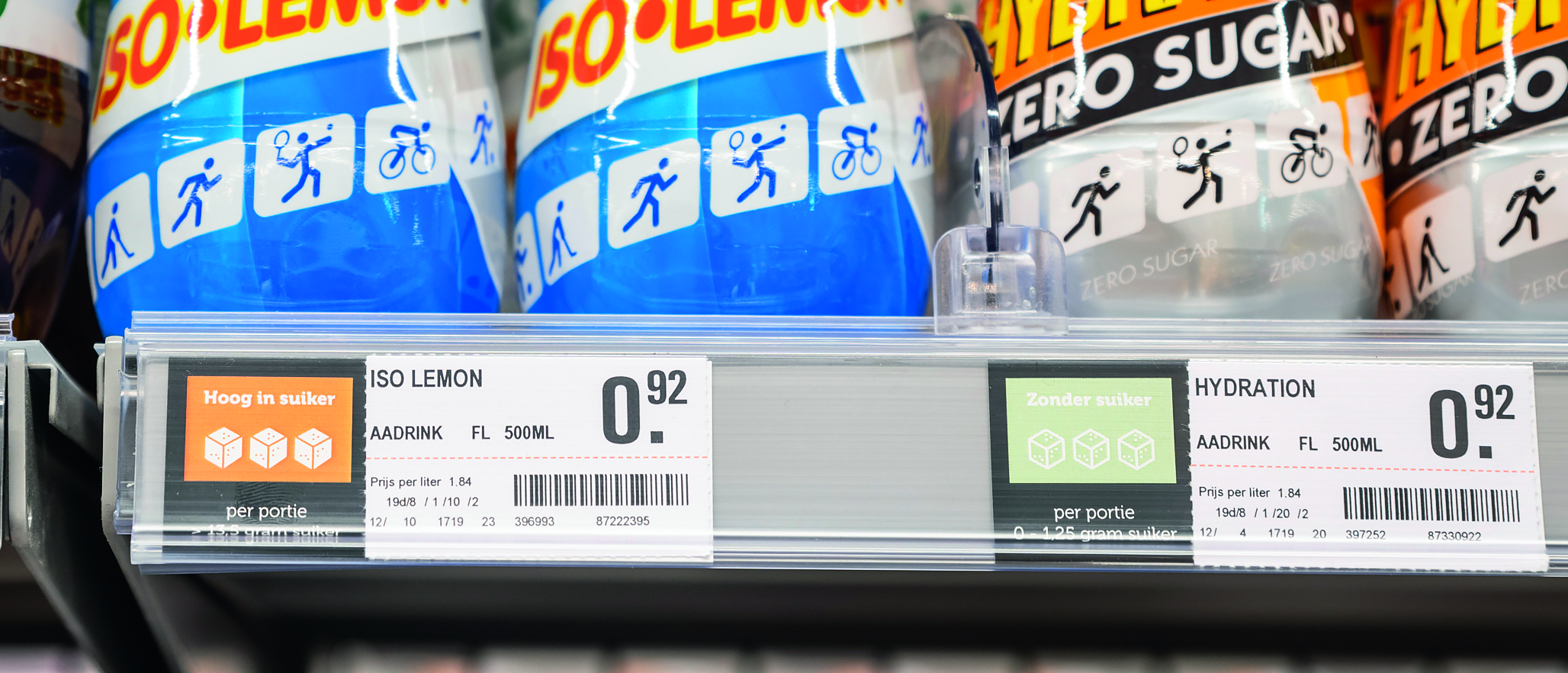


# Supplementary Figure 2. Photograph of the on-shelf sugar labels implemented in store

**
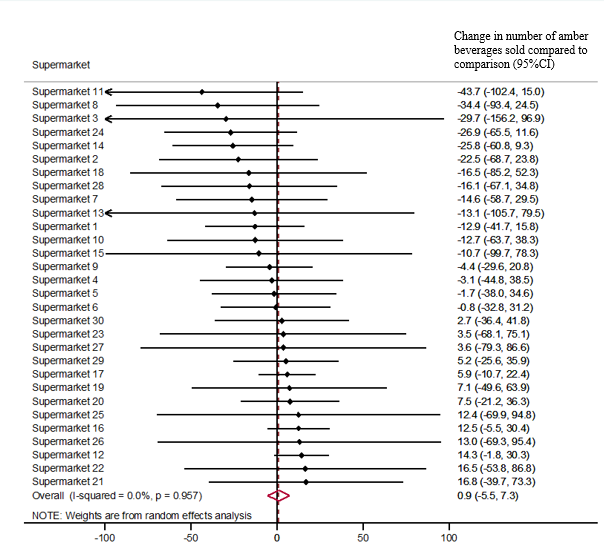
**

# Supplementary Figure 3a. Change in sales of amber beverages compared to comparison stores


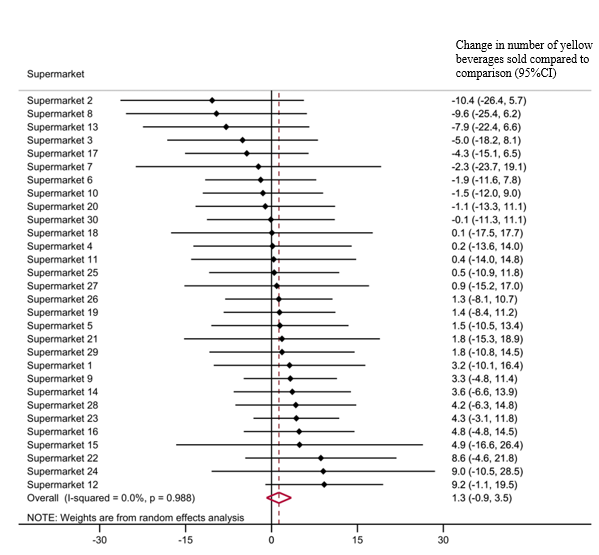


# Supplementary Figure 3b. Change in sales of yellow beverages compared to comparison stores


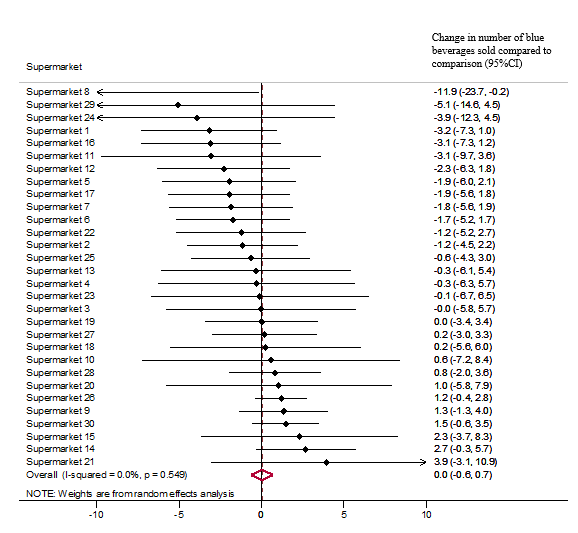


# Supplementary Figure 3c. Change in sales of blue beverages compared to comparison stores


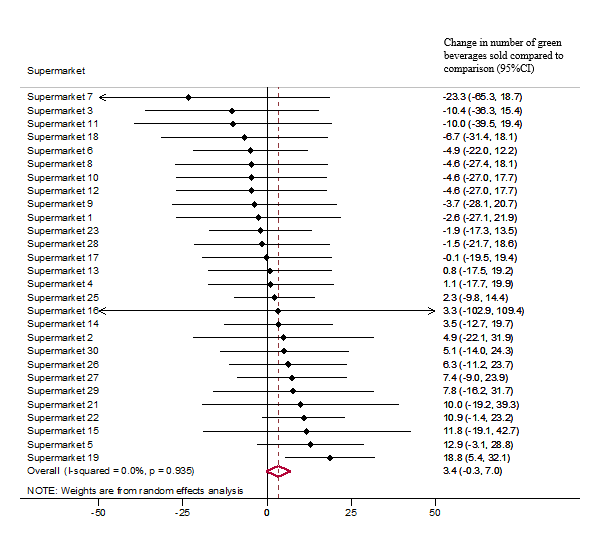


# Supplementary Figure 3d. Change in sales of green beverages compared to comparison stores


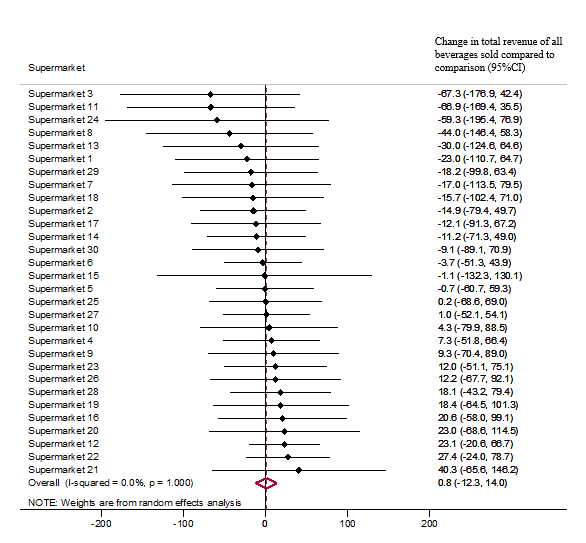


# Supplementary Figure 4. Change in beverage revenue compared to comparison stores


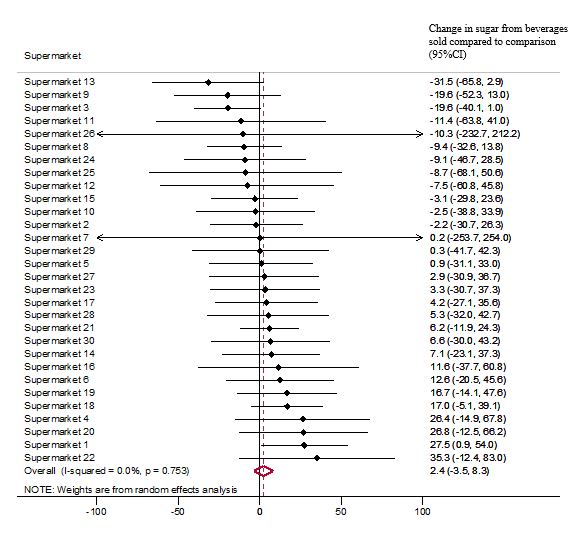


# Supplementary Figure 5. Change in total sugar from beverages sold compared to comparison stores


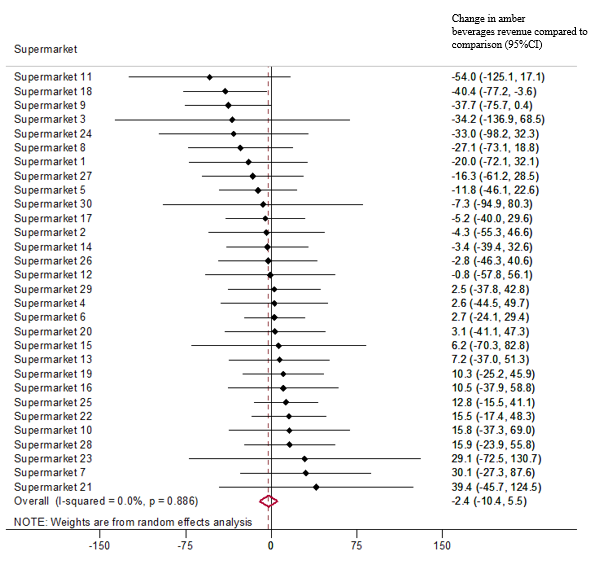


# Supplementary Figure 6a. Change in revenue after implementation of on-shelf sugar labels of amber beverages compared to comparison stores


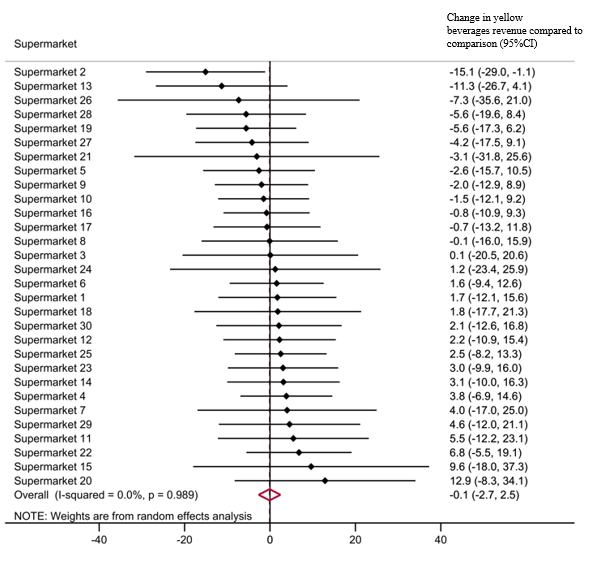


# Supplementary Figure 6b. Change in revenue after implementation of on-shelf sugar labels of yellow beverages compared to comparison stores

**
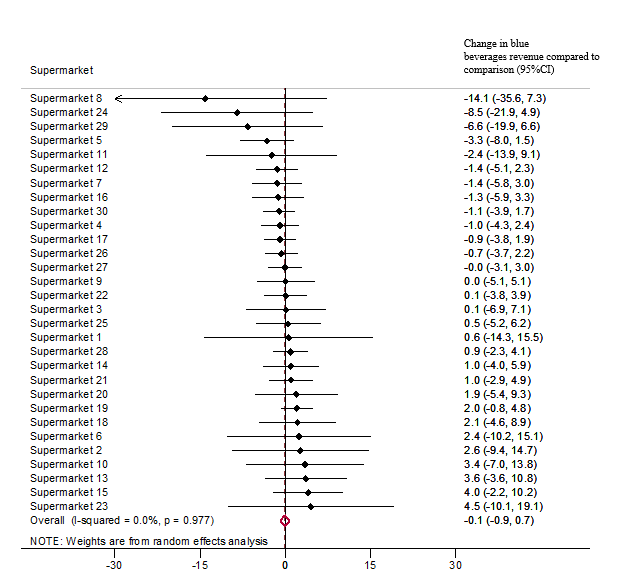
**

# Supplementary Figure 6c. Change in revenue after implementation of on-shelf sugar labels of blue beverages compared to comparison stores

**
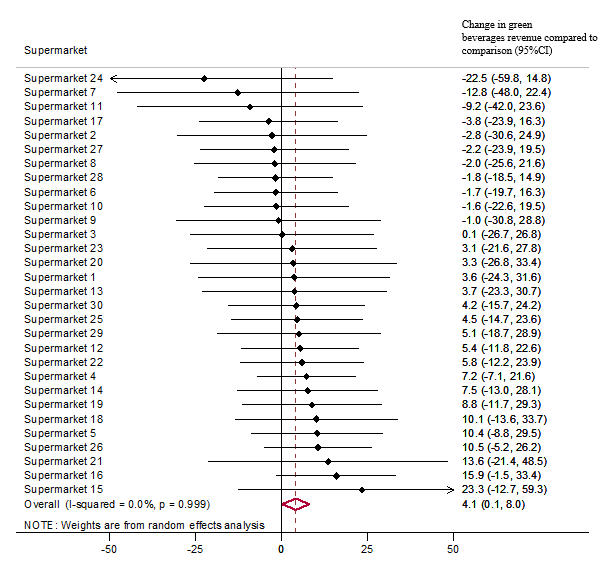
**

# Supplementary Figure 6d. Change in revenue after implementation of on-shelf sugar labels of green beverages compared to comparison stores


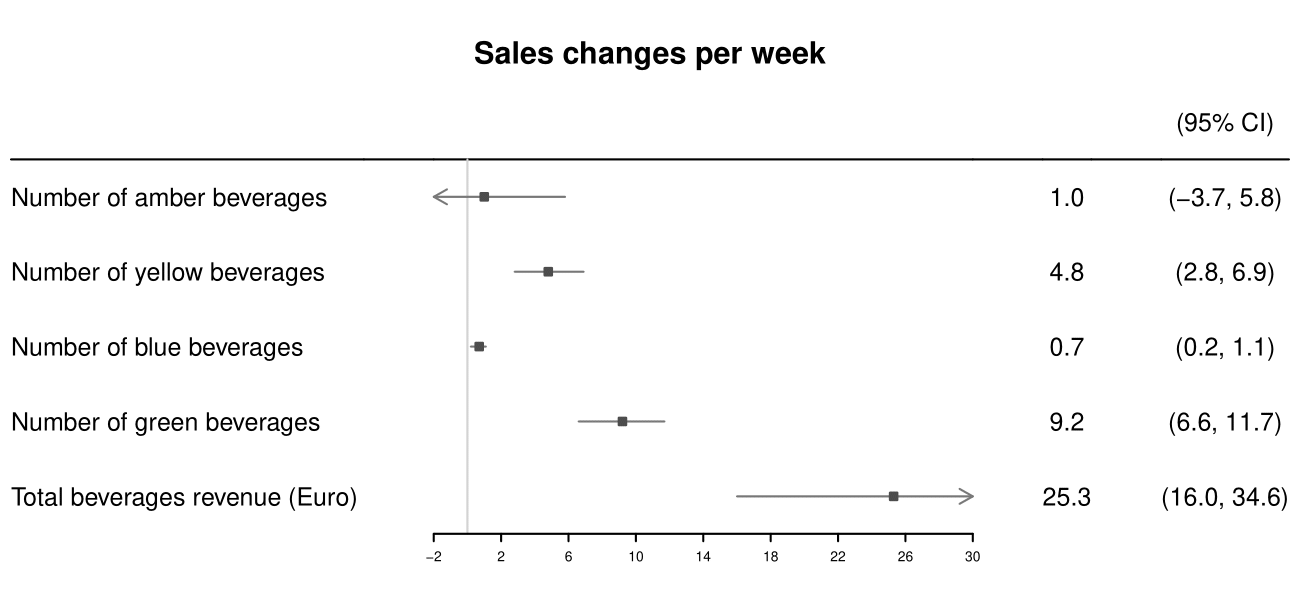


# Supplementary Figure 7. Overall pooled change in sales after implementation of on-shelf sugar labels of amber, yellow, blue and green beverages and change in total beverage revenue


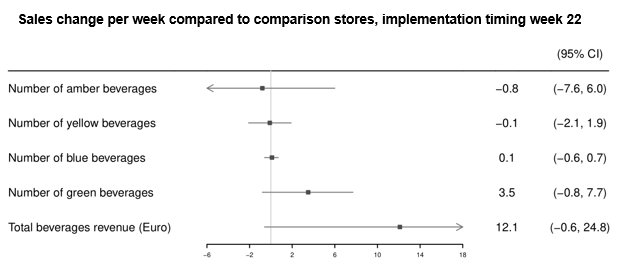


# Supplementary Figure 8. Overall pooled change in sales after implementation of on-shelf sugar labels of amber, yellow, blue and green beverages and total revenue compared to comparison stores, when implementation timing is set at week 22 instead of week 18


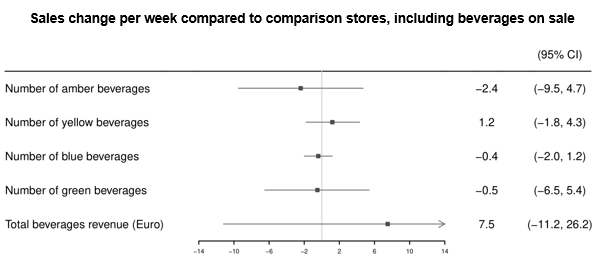


# Supplementary Figure 9. Overall pooled change in sales after implementation of on-shelf sugar labels of amber, yellow, blue and green beverages and total revenue compared to comparison stores, including beverages on sale
